# Acute Supplementation of Soluble Mango Leaf Extract (Zynamite^®^ S) Improves Mental Performance and Mood: A Randomized, Double-Blind, Placebo-Controlled Crossover Study

**DOI:** 10.3390/ph18040571

**Published:** 2025-04-14

**Authors:** Yolanda Castellote-Caballero, Ana Beltrán-Arranz, Agustín Aibar-Almazán, María del Carmen Carcelén-Fraile, Yulieth Rivas-Campo, Laura López-Ríos, Tanausú Vega-Morales, Ana María González-Martín

**Affiliations:** 1 Department of Health Sciences, Faculty of Health Sciences, University of Atlántico Medio, 35017 Las Palmas de Gran Canaria, Spain; 2 Nektium Pharma, Las Mimosas 8, Agüimes, 35118 Las Palmas de Gran Canaria, Spain; abeltran@nektium.com (A.B.-A.); tvega@nektium.com (T.V.-M.); 3 Department of Health Sciences, Faculty of Health Sciences, University of Jaén, 23071 Jaén, Spain; 4 Department of Educational Sciences, Faculty of Social Sciences, University of Atlántico Medio, 35017 Las Palmas de Gran Canaria, Spain; 5 Faculty of Human and Social Sciences, University of San Buenaventura-Cali, Santiago de Cali 760045, Colombia; 6 Department of Psychology, Faculty of Health Sciences, University of Atlántico Medio, 35017 Las Palmas de Gran Canaria, Spain; 7 Department of Psychology, Higher Education Center for Teaching and Educational Research, 28014 Madrid, Spain

**Keywords:** Zynamite, cognitive function, mood, university students, *Mangifera indica*

## Abstract

**Background/Objectives:** A mango (*Mangifera indica*) leaf extract (Zynamite^®^), rich in the polyphenol mangiferin, has been demonstrated to modulate brain activity, boost cognitive function, and reduce mental fatigue. Research evidence supports that improving the solubility of this extract could significantly enhance its efficacy as an active ingredient. This study examined the effects of a soluble version of Zynamite^®^, Zynamite^®^ S (Zyn-S), on cognitive function and mood in young adults at low doses. **Methods:** A total of 119 university students were enrolled in the study. Participants were randomly assigned to receive either 100 mg, 150 mg, or placebo in a double-blind crossover design. Short- and long-term memory were evaluated using the Rey Auditory Verbal Learning Test (RAVLT), executive functions with the Trail Making Test (TMT), processing speed with the Digit Symbol Substitution Test (DSST), and selective attention with the Stroop Color and Word Test. Additionally, mood was assessed using the Spanish short version of the Profile of Mood States (POMS). All these assessments were conducted before taking the product and at 30 min, 3 h, and 5 h post-intake. **Results:** The results demonstrated that participants who received Zynamite^®^ S experienced significant improvements in reduced tension, depression, and confusion, suggesting an enhancement in mental clarity and overall emotional well-being. Both interventions also improved processing speed and cognitive flexibility. However, no significant differences were observed in short- and long-term verbal memory. **Conclusions:** In summary, these findings support Zynamite^®^ S as a natural nootropic capable of acutely improving key cognitive functions and emotional balance at low doses in young adults, with sustained efficacy for at least five hours.

## 1. Introduction

University students frequently face intense academic demands and social transitions, often leading to heightened stress levels that can profoundly impact cognitive performance, particularly affecting attention, executive functions, and fatigue [1]. Acute stress episodes can temporarily disrupt attention and focus, while chronic stress can impair cognitive flexibility and decision making in the long term. Such cognitive disturbances are closely linked to declines in academic performance and personal well-being [2,3]. Furthermore, there is a bidirectional relationship between stress and mood disturbances such as anxiety and depression, which in turn exacerbate cognitive impairments [4]. Developing effective strategies aimed at boosting cognitive functions—such as attention, focus, and executive functions—while simultaneously improving mood is key to supporting academic performance and overall well-being among university students.

Nootropics, also known as “smart drugs”, represent a diverse set of compounds that enhance cognitive functions, such as learning and memory, particularly in environments where these abilities are impaired [5]. The mechanisms of action of nootropics include improving oxygen and glucose supply to the brain, exerting antihypoxic effects, providing protection against neurotoxicity, promoting the synthesis of neuronal proteins and nucleic acids, and stimulating the metabolism of phospholipids in neuroendocrine membranes [6]. In clinical settings, nootropics are prescribed to treat both acute and chronic conditions affecting memory and learning, and they have shown benefits in cases of mild cognitive impairment or memory disorders due to fatigue [7]. Among university students, nootropics have gained popularity as a means to enhance intelligence and cognitive functions [8]. Commonly used nootropics among students include prescription stimulants, such as methylphenidate (Ritalin) and dextroamphetamine/amphetamine combinations (Adderall), which are primarily indicated for attention deficit/hyperactivity disorder (ADHD) but are often used off-label by students seeking improved focus and concentration [9,10]. Additionally, modafinil (Provigil), a wakefulness-promoting agent approved for narcolepsy and other sleep disorders, is utilized for its potential to increase alertness and cognitive performance [11].

Within the category of nootropics, those of natural origin offer a safer side-effect profile and a broader variety of benefits due to potential synergistic or additive effects of their bioactive compounds when compared with synthetic nootropics. Beyond prescription medications, students also use over-the-counter supplements as cognitive enhancers. The most widely used is caffeine, typically consumed via coffee, tea, or energy drinks. Caffeine is sought after for its well-documented effects on alertness, vigilance, and fatigue reduction [12], although consumption in high amounts may lead to side effects, such as jitteriness, nervousness, or insomnia [13]. Alongside caffeine, supplements containing Bacopa monnieri or Ginkgo biloba, or combinations of caffeine and L-theanine, are also used to boost mental energy and performance [14,15,16]. *Mangifera indica* (mango) leaf extract, traditionally used in medicine for its anti-inflammatory, antioxidant, and antidiabetic properties [17], has more recently emerged as a candidate for cognitive enhancement and neuroprotection [18]. The efficacy of mango leaf extracts is primarily attributed to their high concentration of xanthones, particularly mangiferin. In the central nervous system, mangiferin has demonstrated strong antioxidant properties, effectively neutralizing reactive oxygen species (ROS) and reducing oxidative stress [19,20], and it has exhibited anti-inflammatory effects by modulating pro-inflammatory cytokines and suppressing microglial activation [21]. Additionally, mangiferin has shown neuroprotective properties by preventing the depletion of brain-derived neurotrophic factor (BDNF) following exposure to various insults [22,23] and by supporting energy metabolism to protect mitochondrial function [24,25]. Beyond its neuroprotective roles, mangiferin has also been shown to improve learning and memory [26,27,28] and mitigate depressive and anxious behaviors in animal models [29,30].

Several studies have evaluated the potential benefits of a mango leaf extract standardized to contain ≥60% of mangiferin (Zynamite^®^) [31,32,33,34,35,36], observing improvements in physical and cognitive performance. Zynamite^®^ supplementation in combination with additional polyphenols has been demonstrated to increase physical energy by improving mean power output, peak power output, VO_2_ max, brain oxygenation, muscle O_2_ extraction, exercise performance, and muscle recovery and by exhibiting exercise-mimetic properties without side effects [31,32,33,34]. In terms of brain function, Zynamite^®^ has shown to boost mental energy by enhancing attention, improving reaction time, and reducing mental fatigue and stress [35]. Additionally, the extract enhanced the excitability of hippocampal pyramidal cells and modulated brain activity as measured by electroencephalogram (EEG), especially when administered in combination with caffeine [18,36].

Despite the multiple properties attributed to mangiferin, this natural polyphenol has low solubility in most food-grade solvents, which presents a drawback when formulating water-based beverages such as energy drinks and shakes [37]. Moreover, this limited solubility can result in reduced oral bioavailability in humans [38,39]. Along these lines, several studies have demonstrated that increasing mangiferin solubility with different technical approaches, such as the addition of hydroxypropyl-β-cyclodextrin [40] or monosodium salt [41,42], could improve the bioavailability of mangiferin. More recently, the bioavailability of Zynamite^®^ has been increased by incorporating solubilization technologies into its formulation, resulting in a more soluble version of Zynamite^®^, named Zynamite^®^ S (Zyn-S), with up to 2.44 times the bioavailability of mangiferin [43].

Based on the extensive literature supporting the cognitive benefits of Zynamite^®^ and the increased bioavailability of its soluble form (Zyn-S), the aim of the current study was to evaluate the efficacy of a single low dose of Zyn-S on cognitive performance (memory, concentration, attention, and mental agility) and mood (anger, fatigue, vigor, friendliness, tension, and depression) in university students.

## 2. Results

### 2.1. Sociodemographic and Baseline Characteristics of the Subjects

The demographic and anthropometric data of the 119 subjects included in the study are presented in Table 1 by group. The mean age of the study population was 20.26 ± 1.59 years, of whom 49.57% were female. Participants were divided into three groups that were homogeneous in terms of age, gender distribution, treatment start time, physical activity levels, and anthropometric variables, with no significant differences observed between groups.

### 2.2. Cognitive Function Assessments

#### 2.2.1. Short- and Long-Term Memory

**Figure 1:**
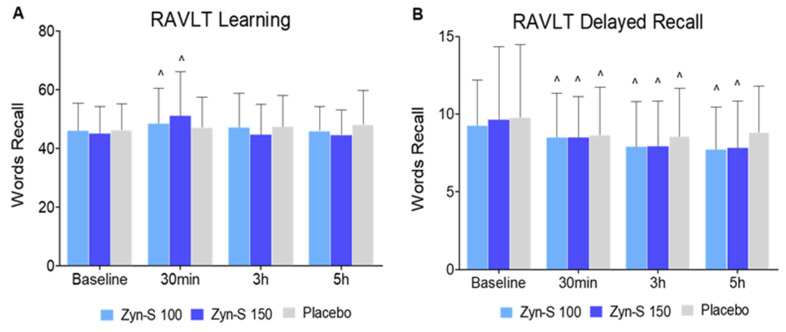
Effects of Zyn-S supplementation on (**A**) RAVLT Learning and (**B**) RAVLT Delayed Recall. Data are shown as means ± SD. Two-tailed Student’s paired *t* test and repeated-measures ANOVA with post hoc Bonferroni adjustment were conducted. ^ = *p* < 0.05 difference from baseline value.

The RAVLT was used to assess verbal memory, including learning efficiency and long-term recall ability. The RAVLT Learning test showed significant improvements at 30 min compared with baseline for both the Zyn-S 100 and Zyn-S 150 groups (*p* = 0.043 and *p* < 0.001, respectively), while the placebo group exhibited no changes over time. The RAVLT Delayed Recall test showed significant declines in the placebo group at 30 min and 3 h (*p* = 0.028 and *p* = 0.014, respectively), whereas the Zyn-S 100 and Zyn-S 150 groups demonstrated significant reductions at 30 min, 3 h, and 5 h post-ingestion (*p* = 0.004 and *p* = 0.011 at 30 min, respectively, and *p* < 0.001 at 3 h and 5 h for both treatments). These findings suggest that Zyn-S treatment enhanced immediate learning but did not sustain its effect over time. Additionally, while Zyn-S supplementation maintained short-term recall comparable to placebo, it improved performance on long-term recall (Figure 1).

The repeated measures ANOVA demonstrated significant effects of Zyn-S interventions on short- and long-term memory. For RAVLT Learning, significant main effects were observed for the treatment time (F(2,234) = 8.97, *p* < 0.001) and the group–time interaction (F(2,234) = 4.74, *p* < 0.001), indicating that the treatment group and its interaction with time significantly influenced learning performance, with variations likely reflecting differences in treatment efficacy over time. In the case of RAVLT Delayed Recall, a significant main effect of time was identified (F(2,234) = 21.65, *p* < 0.001), reflecting substantial changes in recall performance across time points. However, no significant effects were detected for the treatment group (F(2,234) = 1.86, *p* = 0.157) or the interaction between group and time (F(2,234) = 1.28, *p* = 0.264), suggesting that the delayed recall performance was primarily influenced by temporal factors rather than the treatment.

The post hoc analysis did not show any significant differences in learning and delayed recall between groups or over time, suggesting that Zyn-S treatment did not have a significant effect on short- and long-term memory when compared with placebo administration.

#### 2.2.2. Executive Functions

The TMT was used to evaluate multiple cognitive domains, including processing speed, visual attention, and executive function. Statistically significant improvements in cognitive performance, as measured by the TMT-A, were observed exclusively at 5 h for the Zyn-S 100 group (*p* = 0.005) and the Zyn-S 150 group (*p* = 0.003). Similarly, significant improvements were detected in the TMT-B at 5 h for the Zyn-S 100 group (*p* = 0.022) and the Zyn-S 150 group (*p* = 0.001), whereas the placebo group showed no significant change over time.

**Figure 2:**
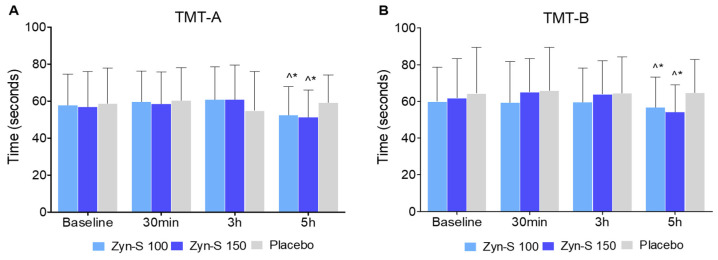
Effects of Zyn-S supplementation on (**A**) TMT-A and (**B**) TMT-B. Data are shown as means ± SD. Two-tailed Student’s paired *t* test and repeated-measures ANOVA with post hoc Bonferroni adjustment were conducted. ^ = *p* < 0.05 difference from baseline value. * = *p* < 0.05 difference compared with placebo group.

The repeated measures ANOVA analysis revealed a significant effect of Time for TMT-A (F(2.234) = 10.46, *p* < 0.001), indicating improvements over time. Additionally, a significant effect on the Group × Time interaction (F(2.234) = 6.70, *p* < 0.001) suggests that the degree of improvement also differed between groups, with the Zyn-S interventions yielding greater benefits compared with the placebo group. However, no significant main effect between the groups was detected (F(2.234) = 0.342, *p* = 0.710), indicating no differences among groups. For TMT-B, significant main effects for Group (F(2.234) = 4.04, *p* = 0.018) and Time (F(2.234) = 9.98, *p* < 0.001), along with a significant Group × Time interaction (F(2.234) = 4.56, *p* < 0.001), demonstrated overall performance improvements over time, with more pronounced reductions in completion time for participants receiving Zyn-S treatment (Figure 2).

The post hoc analysis of the group–time interaction for TMT-A and TMT-B revealed significant differences at 5 h for both Zyn-S 100 and Zyn-S 150. For TMTA, Zyn-S 100 showed a marked reduction in completion time compared with the placebo group (mean difference [MD] = −67.90, *p* = 0.033), and Zyn-S 150 exhibited an even greater improvement (MD = −79.33, *p* = 0.003). In the case of TMTB, Zyn-S 100 significantly outperformed the placebo group (MD = −7.992, *p* = 0.013), while Zyn-S 150 showed the most substantial improvement (MD = −10.588, *p* < 0.001). These findings suggest that Zyn-S treatment improved cognitive speed and visual attention, as demonstrated by performance on TMT-A, and boosted the ability to handle complex tasks, as demonstrated by performance on TMT-B at 5 h post-intervention, with Zyn-S 150 promoting the greatest enhancement in task performance.

#### 2.2.3. Processing Speed

The DSST was employed to measure performance on processing speed, attention, and working memory. Both the Zyn-S 100 and Zyn-S 150 groups exhibited significant increases in DSST scores at all time points. More specifically, the Zyn-S 100 group showed significant reductions at 30 min (67.88 ± 17.05, *p* = 0.007), 3 h (70.19 ± 16.83, *p* < 0.001), and 5 h (72.32 ± 14.35, *p* < 0.001) compared with baseline (64.77 ± 15.39). Similarly, the Zyn-S 150 group demonstrated significant decreases from baseline (60.95 ± 14.42) at 30 min (66.63 ± 13.70, *p* < 0.001), 3 h (68.34 ± 13.23, *p* < 0.001), and 5 h (69.13 ± 13.89, *p* < 0.001). In contrast, the placebo group showed no significant changes at any time point (*p* > 0.05).

**Figure 3:**
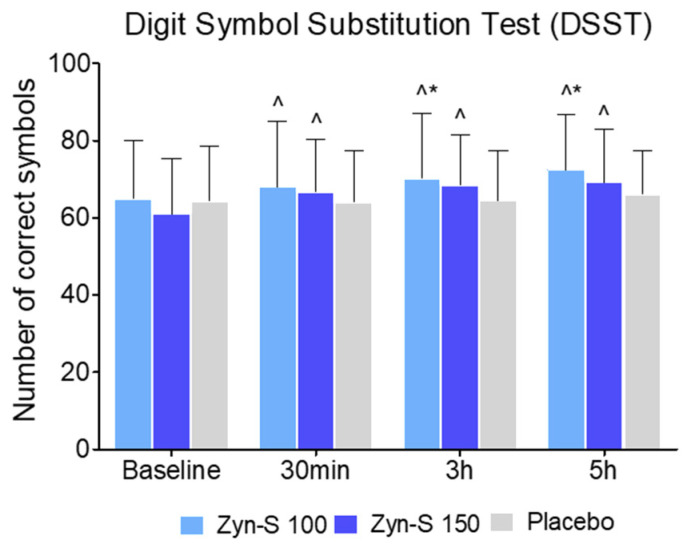
Effects of Zyn-S supplementation on DSST. Data are shown as means ± SD. Two-tailed Student’s paired *t* test and repeated-measures ANOVA with post hoc Bonferroni adjustment were conducted. ^ = *p* < 0.05 difference from baseline value. * = *p* < 0.05 difference compared with placebo group.

The repeated measures ANOVA analysis showed significant main effects for Group (F(2.234) = 3.93, *p* = 0.020) and Time (F(2,234) = 22.03, *p* < 0.001), as well as a significant Group × Time interaction (F(2.234) = 4.02, *p* < 0.001), indicating a consistent increase in performance across time, particularly in the Zyn-S intervention groups, supporting the potential cognitive-enhancing effects of these interventions (Figure 3).

In the post hoc analysis, the Zyn-S 100 treatment showed significant improvement at 3 h over the placebo group (MD = −5.420, *p* = 0.005). This effect became more pronounced at 5 h, with Zyn-S 100 again outperforming the placebo group (MD = −7.546, *p* < 0.001). These results suggest that Zyn-S 100 enhances cognitive performance over time, with the most substantial effects observed at 5 h.

#### 2.2.4. Selective Attention

**Figure 4:**
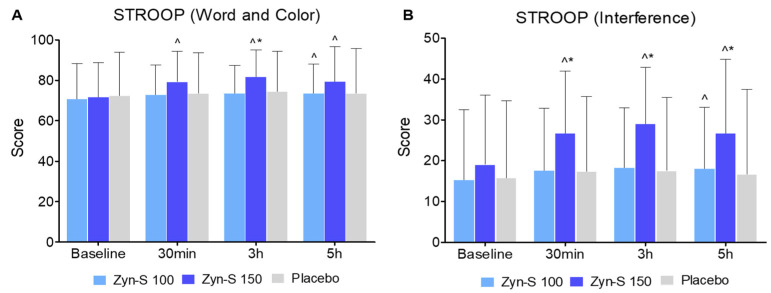
Effects of Zyn-S supplementation on (**A**) Stroop Word and Color (**B**) and Stroop Interference. Data are shown as means ± SD. Two-tailed Student’s paired *t* test and repeated-measures ANOVA with post hoc Bonferroni adjustment were conducted. ^ = *p* < 0.05 difference from baseline value. * = *p* < 0.05 difference compared with placebo group.

The Stroop Color and Word Test was used to assess the cognitive domains of selective attention and executive function. The results showed no significant changes in the placebo group (*p* > 0.05) over time, whereas the Zyn-S 150 group showed significant improvements at all time points (*p* < 0.001), and the Zyn-S 100 group exhibited significant enhancement only at 5 h (*p* = 0.024). The repeated measures ANOVA showed significant main effects for Group (F(2.234) = 4.64, *p* = 0.010) and Time (F(2.234) = 13.03, *p* < 0.001), together with a significant Group × Time interaction (F(2.234) = 3.64, *p* < 0.001), highlighting improvements in Stroop performance, with greater enhancements in the Zyn-S groups compared with the placebo group. In the post hoc analysis, no significant differences were found between the groups at baseline or at 30 min post-intervention (*p* > 0.05). At 3 h, Zyn-S 150 exhibited a significant improvement compared with both Zyn-S 100 (MD = −812.605, *p* = 0.007) and the placebo group (MD = −719.328, *p* = 0.038). By 5 h, no significant group differences were detected (*p* > 0.05), indicating that the improvements in selective attention after Zyn-S 150 administration were transient, peaking at 3 h post-intervention (Figure 4).

The Stroop interference test, which measures cognitive flexibility, revealed that Zyn-S 150 treatment improved cognitive performance at all time points (*p* < 0.001), whereas the Zyn-S 100 group showed significant improvements only at 5 h (*p* = 0.025). No significant changes were detected in the placebo group (*p* > 0.05). The repeated measures ANOVA showed significant main effects for Group (F(2,234) = 14.2, *p* < 0.001) and Time (F(2,234) = 12.95, *p* < 0.001), along with a significant Group × Time interaction (F(2,234) = 3.53, *p* = 0.002), highlighting improvements in Stroop performance over time and among groups, with greater improvements in the Zyn-S groups compared with the placebo group. The post hoc analysis revealed that Zyn-S150 showed significant improvements in cognitive flexibility at 30 min compared with Zyn-S-100 (MD = −9.072, *p* = 0.002) and the placebo group (MD = −9.334, *p* ≤ 0.001), as well as at 3 h (MD = −10.707, *p* ≤ 0.001; MD = −11.478, *p* ≤ 0.001) and at 5 h (MD = −8.6413, *p* ≤ 0.001; MD = −10.051, *p* = 0.002).

### 2.3. Emotional State Assessments

The POMS test evaluates several domains related to mood and emotional state. The results from this assessment revealed significant improvements across the tension, depression, and confusion domains as well as for total mood disturbance scores at most time points for both Zyn-S 100 (*p* < 0.05) and Zyn-S 150 (*p* < 0.001) groups. In contrast, no significant changes were observed in the placebo group over time (*p* > 0.05).

Regarding the tension domain, the repeated measures ANOVA analysis revealed significant main effects for Group (F(2,234) = 8.0, *p* < 0.001) and Time (F(2,234) = 72.3, *p* < 0.001), along with a robust Group × Time interaction (F(2,234) = 20.7, *p* < 0.001), indicating substantial reductions in tension over time, especially among participants that received Zyn-S treatments. No significant group differences were observed at baseline and at 30 min in the post hoc analysis (*p* > 0.05). However, a significant mean difference was observed between the placebo and Zyn-S 150 groups at 3 h (MD = 0.222, *p* = 0.001). At 5 h, both Zyn-S 100 (MD = −0.263, *p* = 0.001) and Zyn-S 150 (MD = 0.390, *p* = 0.001) showed significant differences compared with the placebo group.

For depression, although no significant main effect for Group was detected (F(2.234) = 0.915, *p* = 0.401), significant effects for Time (F(2.234) = 54.73, *p* < 0.001) and the Group × Time interaction (F(2.234) = 9.14, *p* < 0.001) suggest that reductions in depression scores were driven primarily by temporal changes, with greater improvements in the Zyn-S groups. The post hoc analysis showed no significant differences between groups at baseline, 30 min, 3 h, and 5 h.

In the case of confusion, significant main effects for Group (F(2.234) = 14.8, *p* < 0.001) and Time (F(2.234) = 90.8, *p* < 0.001), as well as a significant Group × Time interaction (F(2.234) = 18.2, *p* < 0.001), underscored marked reductions in confusion and increased mental clarity, particularly in participants receiving Zyn-S interventions. The post hoc analysis showed no significant differences at baseline between groups. At 30 min, the Zyn-S 100 group showed a non-significant trend compared with placebo (MD = −1.798, *p* = 0.052), whereas at 3 h, a significant difference was observed between Zyn-S 100 and placebo (MD = −2.630, *p* < 0.001), with no significant differences for comparisons between other groups. At 5 h, both Zyn-S 100 vs. placebo (MD = −4.471, *p* < 0.001) and placebo vs. Zyn-S 150 (MD = 2.782, *p* < 0.001) were significant, with Zyn-S 100 vs. Zyn-S 150 also reaching a significant difference (MD = −1.689, *p* = 0.005).

**Figure 5:**
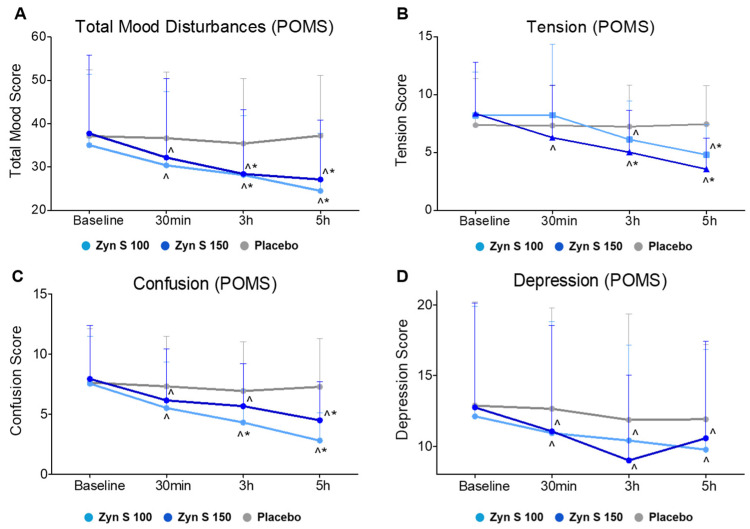
Effects of Zyn-S supplementation on multiple domains of POMS: (**A**) Total Mood Disturbances, (**B**) Tension, (**C**) Confusion, and (**D**) Depression. Data are shown as means ± SD. Two-tailed Student’s paired *t* test and repeated-measures ANOVA with post hoc Bonferroni adjustment were conducted. ^ = *p* < 0.05 difference from baseline value. * = *p* < 0.05 difference compared with placebo group. For the rest of the emotional states evaluated (anger, fatigue, and vigor), no significant changes were observed, neither at different times compared with the baseline nor improvements between groups.

For the POMS total score, significant effects were observed for Group (F(2.234) = 7.64, *p* < 0.001), Time (F(2.234) = 88.6, *p* < 0.001), and the Group x Time interaction (F(2.234) = 20.8, *p* < 0.001), suggesting that the pattern of change in POMS scores over time differed significantly between groups. In the post hoc analysis, significant improvements were noted at 3 h for Zyn-S 100 vs. placebo (*p* = 0.009) and placebo vs. Zyn-S 150 (*p* = 0.014), with no significant difference between Zyn-S 100 and Zyn-S 150. By 5 h, both Zyn-S 100 and Zyn-S 150 showed significant improvements compared with placebo (*p* < 0.001 for both), while differences between Zyn-S 100 and Zyn-S 150 remained non-significant. These results highlight the sustained mood-enhancing effects of Zyn-S formulations (Figure 5).

## 3. Discussion

Mangiferin, a xanthone compound derived from mango leaves, exhibits a broad spectrum of biological properties, making it a promising candidate for various therapeutic applications. Antioxidant, anti-inflammatory, anticancer, antidiabetic, and cardiovascular protective effects are well documented for mangiferin [44,45]. Notably, mangiferin’s cognitive-enhancing effects have garnered attention in recent years [18]. A clinical study investigated the acute cognitive effects following the administration of 300 mg of Zynamite^®^, a standardized mango leaf extract containing 60% mangiferin [35]. Employing a double-blind, placebo-controlled, crossover clinical study in 70 healthy young adults (18–45 years), the research demonstrated significant improvements, primarily in the accuracy of cognitive performance across a comprehensive battery of tasks (COMPASS). These benefits were particularly evident in specific cognitive domains, with significant enhancements observed on sustained attention, working memory, and executive function. Notably, these cognitive benefits were not transient, as they were observed from 30 min up to 5 h after administration, indicating a sustained effect over a significant period. Moreover, the synergistic effects of this extract with other polyphenols have demonstrated benefits beyond cognition, including enhanced athletic performance, reduced muscle damage, and accelerated recovery [31,32,33,34].

Despite numerous beneficial properties, the therapeutic potential of mangiferin is hampered by its poor bioavailability, largely attributed to its low solubility. Thus, high doses of mangiferin are required to achieve notable effects. To address this challenge, recent research developed a water-soluble monosodium salt version of a standardized mango leaf extract, named Zynamite^®^ S. A human pharmacokinetic study demonstrated a 2.44-fold increase in Zynamite^®^ S bioavailability compared with the standard non-soluble extract (Zynamite^®^) [43], potentially enabling lower effective doses.

The current study evaluated the effects of oral intake of a single dose of Zynamite^®^ S (100 mg or 150 mg) compared with placebo on cognitive function and mood in university students, revealing promising outcomes across several cognitive and emotional domains. This study significantly advances our understanding of Zynamite^®^ S potential as a nootropic ingredient, offering distinct novel insights compared with previous research. A primary contribution is the demonstration that Zynamite^®^ S elicits significant improvements across multiple cognitive domains—including processing speed, selective attention, and executive functions—at considerably lower acute doses (100 mg and 150 mg) than the 300 mg dose of non-soluble Zynamite^®^ previously documented to enhance cognition [35]. This finding strongly supports the advantage conferred by the enhanced bioavailability of Zyn-S [43], indicating greater physiological efficiency and potentially allowing for lower consumer intake levels. Importantly, this research also provides strong evidence that Zynamite^®^ S can significantly improve aspects of emotional state concurrently with cognitive performance. Whereas prior studies with non-soluble Zynamite^®^ reported inconclusive or no significant effects on mood [35], the current findings showed marked reductions in negative states such as tension and confusion, suggesting an improved overall well-being. Therefore, Zynamite^®^ S emerges as a more dose-efficient cognitive enhancer due to its enhanced solubility, and it uniquely offers valuable dual benefits by simultaneously supporting cognitive function and promoting a more positive emotional state, highlighting its potential relevance for managing demanding situations involving both cognitive load and stress.

### 3.1. Impact on Cognitive Function

The effects of Zynamite^®^ S (Zyn-S) were evaluated at doses of 100 mg and 150 mg compared with placebo on various cognitive domains, including memory (RAVLT), cognitive flexibility (TMT), processing speed (DSST), and attention/inhibition of automatic responses (Stroop test). Consistent with previous findings from a clinical study on 300 mg of a mango leaf extract standardized to 60% mangiferin (Zynamite^®^) [35], we now observed that acute administration of just 100 mg and 150 mg of Zyn-S led to significant improvements in key cognitive functions, including information processing speed, cognitive flexibility, and attention. Thus, the present study supports our hypothesis that low doses of Zynamite^®^ S enhanced cognitive performance and decision-making processes.

Interestingly, dose-dependent effects of Zynamite^®^ S were observed in different cognitive tasks. While the 100 mg dose was sufficient to address moderate cognitive demands like the DSST, the 150 mg dose yielded superior performance improvements, particularly in tasks requiring greater cognitive control and inhibition of automatic responses, like the Stroop test. The cognitive-enhancing activities of Zyn-S may be partly attributed to the partial inhibition of catechol-O-methyltransferase (COMT) by mangiferin [18]. COMT is an enzyme that catalyzes the methylation of catechol substrates, like dopamine, and plays a crucial role in regulating dopamine levels in the prefrontal cortex. The modulation of dopamine levels in the prefrontal cortex of the brain enhances cognitive functions such as executive control, working memory, and attentional control [45]. Previous studies have shown that mangiferin partially inhibits COMT [18]. At lower doses, Zynamite^®^ S, which is standardized at 60% of mangiferin, likely causes a modest increase in dopamine, sufficient to enhance performance on low-demanding tasks that require moderate cognitive engagement, such as sustained attention and processing speed, as revealed by the Stroop Color and Word and DSST tests. Conversely, higher doses of the extract may result in a more substantial increase in dopamine through greater COMT inhibition [46], benefiting high-demanding tasks like managing cognitive interference and executive functioning, as measured through Stroop interference and TMT tests, which require robust dopaminergic signaling to meet heightened cognitive demands [47].

The cognitive-enhancing effects promoted by Zynamite^®^ S supplementation could also be explained by mangiferin’s ability to inhibit acetylcholinesterase (AChE) [22,48], the enzyme responsible for breaking down acetylcholine in the synaptic cleft. Acetylcholine plays a crucial role in brain function by modulating various cognitive domains, including attention, learning, and memory [49,50], and its depletion is linked to cognitive decline in aging [51]. By inhibiting AChE, mangiferin could increase acetylcholine availability, thereby enhancing cholinergic neurotransmission and supporting cognitive functions.

### 3.2. Mood and Stress Perception Improvements

The impact of Zynamite^®^ S on improving negative mood states was assessed using the Profile of Mood States (POMS) test, a widely recognized tool for measuring transient mood states, psychological distress, and emotional well-being across multiple dimensions. This study demonstrated that Zynamite^®^ S treatment was effective in improving negative mood states across tension, confusion, depression, and overall score dimensions compared with placebo. A previous study in which 300 mg of Zynamite^®^ was acutely administered reported benefits primarily linked to episodic memory and attention tasks, with no significant impact on mood noted [35]. In contrast, the present study supported positive mood effects obtained with acute supplementation of only 100 mg and 150 mg Zyn-S, underscoring its potential for contributing to both cognitive and emotional health.

While administration of a lower dose of Zynamite^®^ S (100 mg) appears to be sufficient to achieve significant improvement in all dimensions, particularly in POMS total score and confusion, the higher dose (150 mg) offers greater improvement in the tension score but does not significantly differ from the effects observed with the 100 mg dose. In addition, the greatest improvements in POMS scores occurred at 3 and 5 h, suggesting that acute administration of Zynamite^®^ S has a gradual onset of action and a positive effect on mood that is sustained for at least five hours.

### 3.3. Cognitive and Emotional Interactions for Practical Applications

The combined cognitive and mood-enhancing effects of Zynamite^®^ S provide a comprehensive mental performance benefit profile, especially in scenarios where individuals face high cognitive and emotional demands, such as the academic environment experienced by university students [52,53]. Academic life often requires sustained attention, rapid processing of information, and the ability to switch flexibly between tasks, all while managing deadlines, examinations, and social pressures. These demands can lead to heightened stress, emotional distress, and cognitive fatigue, which can compromise both academic performance and overall emotional well-being [54].

Zynamite^®^ S’s ability to enhance cognitive flexibility, processing speed, and attention directly addresses these challenges, supporting students’ capacity to manage complex workloads and perform effectively under pressure. Moreover, the observed reductions in emotional distress may work synergistically with cognitive benefits to foster greater emotional resilience [55,56]. A reduction in negative mood states, such as tension and confusion, could help alleviate the cognitive interference caused by stress, further enhancing mental clarity and productivity. This dual action highlights Zynamite^®^ S’s potential as a nootropic to support both cognitive performance and emotional state, particularly in populations exposed to demanding mental and emotional events. In addition to students, it could also be relevant for individuals facing high-stress situations, such as over-busy parents and professionals in demanding, high-pressure jobs.

While the 100 mg dose of Zynamite^®^ S effectively improves mood and stress perception across multiple parameters, the 150 mg dose may be more suitable for more sustained or intense cognitive demands, where prolonged effects of up to five hours are needed.

### 3.4. Limitations and Future Directions

Although this study yielded significant positive results, several limitations must be considered when interpreting the findings. One major limitation of this study is the inter-individual variability among participants, which consisted of a group of university students during exam period. Previous pharmacokinetic studies have reported high inter-individual variability in the absorption of mangiferin [43]. In addition, physiological and genetic differences such as baseline neurotransmitter levels, or different psychological predispositions like motivation or anticipation of reward for good results, can influence outcomes in the clinical trial. Although the study aimed to recruit a large number of participants to minimize inter-individual variations, the heterogeneity in the response could result in masking Zyn-S health benefits for specific population groups. Future research with longitudinal designs and greater control of individual variables is needed to better understand these complex interactions.

A second limitation of this study is its acute design, wherein cognitive function and performance were assessed following a single administration of Zyn-S. While our results provide insights into the short-term effects, the extent to which these effects persist or accumulate with repeated Zyn-S administration remains unknown. Long-term studies are necessary to determine whether chronic supplementation leads to sustained and consistent cognitive benefits. An additional limitation of the present study is the absence of direct neurobiological measures, preventing an elucidation of the molecular mechanisms underlying the effects observed with Zynamite^®^ S. It is well established that key neurotransmitter systems are crucial for mediating cognitive functions and emotional states. For instance, catecholamines like dopamine and norepinephrine are critical for regulating executive functions, attention, and processing speed, particularly within prefrontal cortex circuits [57,58]. While our findings demonstrate significant functional improvements in these cognitive domains following Zyn-S administration, the lack of direct neurochemical measurement prevents us from pinpointing the pathways being modulated. Although exploring neurotransmitter measurements was beyond the scope of the current study design, the precise neurobiological underpinnings of Zynamite^®^ S’s acute effects remain to be directly investigated in future studies. Finally, this study examined cognitive effects from only two low Zyn-S doses (100 and 150 mg). Evaluating a broader dose range, particularly higher doses, might yield a more pronounced cognitive impact or influence the onset time of the response. Therefore, future research incorporating a wider dose-ranging design is necessary to fully characterize the dose–response profile for Zyn-S and to determine the optimal dosage for maximizing cognitive benefits.

## 4. Materials and Methods

### 4.1. Study Design

This clinical trial was conducted at a University Clinical Research facility as a double-blind, randomized, placebo-controlled, crossover study, where the acute effects of a single dose of mango leaf extract (100 mg or 150 mg) and placebo were evaluated on cognitive performance and emotional state at 30 min, 3 h, and 5 h post-administration. Given the ~2.5-fold higher bioavailability of Zyn-S [43], we tested doses of 100 mg and 150 mg, representing approximately 1/3 and 1/2, respectively, of the 300 mg Zynamite^®^ dose known to enhance cognitive performance [35]. The study was retrospectively registered (NCT06651710) and approved by the Ethics Committee of the University of Atlántico Medio (CEI/05-009).

### 4.2. Participants

A total of 119 healthy subjects aged between 18 and 25 years, who were enrolled in an undergraduate or graduate program at university and were free from any relevant medical condition or disease, participated in the study. The exclusion criteria were: (i) having chronic diseases such as asthma, type 1 diabetes, thyroid disorders (e.g., hypothyroidism or hyperthyroidism), autoimmune disorders (e.g., Crohn’s disease), and psychiatric conditions such as anxiety disorders, major depression, eating disorders (anorexia, bulimia), attention deficit/hyperactivity disorder (ADHD), among others; (ii) being pregnant or breastfeeding; (iii) having a known intolerance, hypersensitivity, or allergy to any of the ingredients of the investigational products; (iv) having intestinal absorption problems, learning difficulties, dyslexia, visual problems, or color blindness; (v) taking psychoactive substances or medications that might influence study results, such as consuming >500 mg of caffeine (>6 150 mL cups of brewed coffee) and/or alcohol per day; and (vi) having comprehension or communication difficulties that would prevent full understanding of the informed consent or effective participation in the study, such as autism spectrum disorders, aphasia, or intellectual disabilities.

### 4.3. Randomization

**Figure 6:**
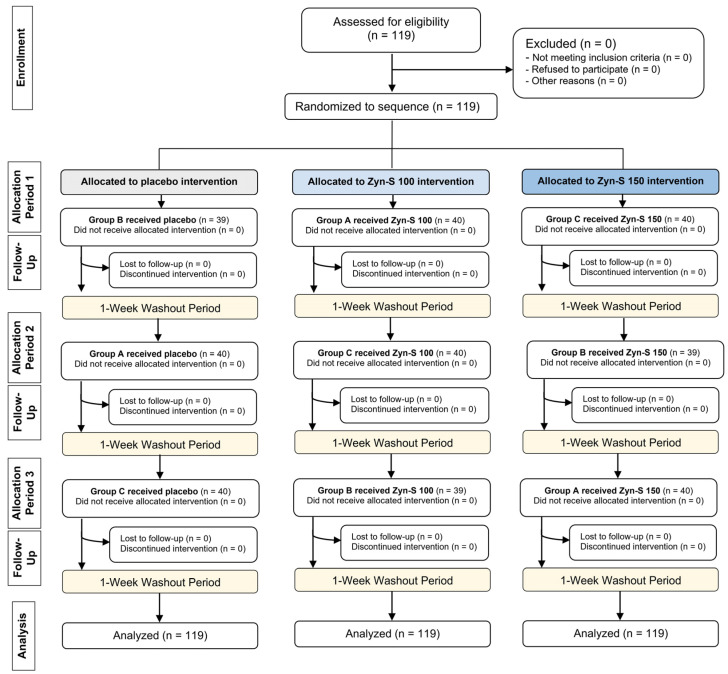
CONSORT flow diagram illustrating the flow of participants through the phases of the study and the number that withdrew at each time point.

Participants were randomly assigned and matched by sex into three groups (Figure 1). Each group received a different treatment on each experimental day. The treatments were placebo, 100 mg of soluble Zynamite^®^ (Zyn-S 100), or 150 mg of soluble Zynamite^®^ (Zyn-S 150). Accordingly, each participant received the three different supplements at different time periods throughout the study. The interval between the test days was 7 days, corresponding to the washout period between treatments. Both participants and researchers were blinded to the treatment assignment, with only the person responsible for the randomization and the statistician being aware of the treatment distribution (Figure 6).

### 4.4. Supplementation Protocol

Participants consumed one capsule per day of either a placebo, consisting of 364 mg of maltodextrin, or one of the two doses of soluble mango (*Mangifera indica)* leaf extract (Zyn-S) standardized to contain 60% of mangiferin (Nektium Pharma, Las Palmas de GC, Spain). The doses consisted of 100 mg of Zyn-S, containing 60 mg of mangiferin (Zyn-S 100), or 150 mg of Zyn-S, containing 90 mg of mangiferin (Zyn-S 150). The supplements or placebo were administered in opaque gelatin capsules (size 00) along with a glass of water. On test days, the supplements were administered 30 min after the first assessment. The study employed a double-blind design to ensure that both participants and researchers were unaware of the treatment allocation.

### 4.5. Procedure

Participants attended the university on a total of four occasions: days 0, 1, 7, and 14. The test days were separated by a 7-day washout period. On each visit, cognitive function markers and emotional states/mood scores were collected 30 min before supplement intake and at 30 min, 3 h, and 5 h post-ingestion.

Screening and training (Day 0): Participants provided informed consent and were screened for eligibility based on inclusion and exclusion criteria. Sociodemographic and clinical data, including age, sex, height, weight, waist-to-hip ratio, and Body Mass Index (BMI) were collected. BMI was calculated as weight (kg) divided by height squared (m^2^). At the end of the session, participants received instructions for the upcoming test visits.

**Figure 7:**
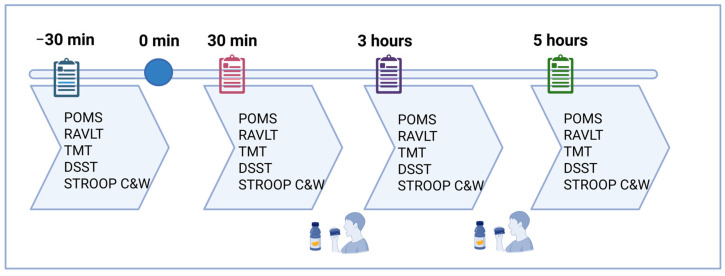
Flow chart of the study procedures throughout the test days. POMS: Profile of Mood States. RAVLT: The Rey Auditory Verbal Learning Test. TMT: Trail Making Test. DSST: Digit Symbol Substitution Test. STROOP: Stroop Color and Word Test and Stroop Interference.

Test days (Day 1, Day 7, and Day 14): Participants arrived at a consistent time in the morning, abstaining from alcohol for 24 h and caffeine from the night before, and consuming a light breakfast no later than one hour prior to arrival. Adherence to these conditions was confirmed on arrival. Once at the facility, participants were restricted to water intake and study-provided food. Test instructions were reviewed, and baseline questionnaires were completed 30 min before receiving the first treatment (placebo, 100 mg of Zyn-S, or 150 mg of Zyn-S). Treatments were administered in a double-blind manner. Follow-up questionnaires were completed 30 min post-ingestion, accompanied by juice and digestive biscuits. After 3 h, participants completed additional questionnaires and consumed a standardized lunch (cheese sandwich, potato chips, and custard/yogurt). Questionnaires were completed again at 5 h post-ingestion. (Figure 7).

### 4.6. Outcomes

#### 4.6.1. Profile of Mood States

Participants completed the Spanish short version of the Profile of Mood States (POMS) 65-item questionnaire [59], which includes a total of 6 subscales: tension (score range 0 to 36), depression (score range 0 to 60), anger (score range 0 to 48), fatigue (score range 0 to 28), vigor (score range 0 to 32), and confusion (score range 0 to 28), as well as a total mood disturbance score (score range −32 to 200). Each of the questions had a score ranging from 0 (not at all) to 4 (very much). The total mood disturbance score was calculated by summing the five negative subscale scores (tension, depression, anger, fatigue, and confusion) and subtracting the vigor score. Higher scores for the total mood disturbance indicate a greater degree of mood disturbance.

#### 4.6.2. Executive Functions

The Trail Making Test (TMT) [60] specifically measures timed motor and visual tasks and was therefore used to assess executive function. This test is divided into two parts: Part A (TMT-A), which evaluates psychomotor attention and speed and involves connecting consecutively numbered circles, and Part B (TMT-B), which requires connecting alternating circles of letters and numbers and is used to measure executive function. A longer time taken to complete the test is interpreted as poorer performance.

#### 4.6.3. Processing Speed

The Digit Symbol Substitution Test (DSST) [61] is a paper-and-pencil cognitive test presented on a single sheet of paper, requiring the subject to match symbols with numbers according to a key located at the top of the page. The subject copies the symbol into spaces beneath a row of numbers. The score is based on the number of correct symbols the participant manages to substitute within 90 s.

#### 4.6.4. Selective Attention

The Stroop Color and Word Test [62] was used to assess selective attention. This test consists of three sheets to complete, each with a duration of 45 s, making the total time for the entire test 5 min. To read each sheet correctly, it should be read from top to bottom and from left to right. The first sheet (Stroop Word) contains 100 words, with the words “RED”, “BLUE”, and “GREEN” repeated in random order and printed in black ink. This part evaluates the number of words read in 45 s, and after this time, participants must circle the last word they read. The second sheet (Stroop Color) consists of 100 identical “XXXX” elements in blue, red, and green ink. The participant must name the color of “XXXX” as quickly as possible. Finally, the third sheet (Stroop Word-Color) contains 100 words referring to a color but printed in a different color. Participants must say the color of the ink in which each word is written. The time taken to complete each stage was measured with a stopwatch. As for scoring, the “P” refers to the number of words read during the first sheet; the “C” refers to the number of colors named; and “PC” refers to the number of elements named in the third sheet. A T score of interference (Stroop Interference) is then calculated based on performance in all three conditions, with higher scores indicating better performance.

#### 4.6.5. Short- and Long-Term Memory

The Rey Auditory Verbal Learning Test (RAVLT) [63] is a neuropsychological test used to assess short- and long-term memory. It comprises two key elements: (i) Learning: Participants are read a list of 15 unrelated words (List A) in five consecutive trials. After each trial, they are asked to repeat all the words they remember. (ii) Delayed Recall: After a time interval without practicing List A (usually 20–30 min, during which participants perform other unrelated tasks), participants are asked to recall and repeat the words from List A again. The scoring criteria for the test include the following: (a) Total learning score: The total number of words correctly recalled in the five trials of List A. Normal scores include recalling 6 words correctly on the first trial and 12–13 on the fifth trial. This reflects short-term learning and memory. The score can range from 0 to 75 points. (b) Delayed Recall: The number of words recalled from List A after the time interval without practice (20–30 min) is noted. This score assesses long-term memory and can range from 0 to 15 points.

### 4.7. Statistical Analysis

The sample size calculation was performed with a 95% confidence level and a 90% power for a randomized, controlled, crossover clinical trial. A minimum clinically significant difference of 2.5 points in the mean of the data was established. Using the critical values from the standard normal distribution for the confidence level (approximately 1.96) and desired power (approximately 1.28), along with an estimated population variance of 1.9, a sample of 99 people was calculated. After accounting for a 15% attrition rate, the required sample size was adjusted to 114 participants, divided into three groups of 38 participants each. This controlled clinical trial employed a crossover design to yield comprehensive estimates of the treatment effects across the three groups. Participants were randomized into treatment groups using a balanced Latin Square design program (https://www.random.org/strings/ (accessed on 2 February 2025)).

In this controlled clinical trial, a crossover design was employed to provide comprehensive estimates of the treatment effects across three groups (Zyn-S 100, Zyn-S 150, and placebo). The demographic and clinical characteristics of the participants were described using descriptive statistics, with means and standard deviations calculated for continuous variables and frequencies and percentages reported for categorical variables. The normality requirements of the population sample were evaluated using the Shapiro–Wilk test. A paired *t* test was used to assess changes over time within each treatment group, providing insights into intra-group differences across the evaluation time points. Subsequently, a repeated-measures ANOVA was conducted to examine the main effects of time, group, and the group × time interaction. This analysis allowed for the assessment of between-group differences and time-dependent treatment effects. Post hoc analyses with Bonferroni adjustment were performed to correct for multiple comparisons, ensuring control of Type I error and a robust interpretation of the results. Statistical analyses were conducted using Stata 14, leveraging its advanced capabilities for implementing mixed models and repeated-measures designs. The results were presented as point estimates with corresponding 95% confidence intervals. To ensure the validity and reproducibility of the findings, all statistical procedures were reviewed by an independent biostatistician. This comprehensive analytical approach enabled a thorough and reliable evaluation of the effects observed in this crossover clinical trial.

## 5. Conclusions

The results confirm that the acute administration of low doses (100 mg and 150 mg) of a soluble extract of *Mangifera indica* (60% mangiferin), known as Zynamite^®^ S, promoted significant improvements in cognitive function tasks related to selective attention, processing speed, and executive functions, with a dose response where 150 mg of Zynamite^®^ S yielded superior performance improvements, particularly in tasks requiring greater cognitive control and inhibition of automatic responses. Moreover, there was a notable reduction in negative emotional states, such as confusion and tension, with an overall positive impact on emotional well-being. These effects were most pronounced at 3 and 5 h post-intervention, suggesting a sustained duration of action.

This study provides compelling evidence that Zynamite^®^ S not only enhances cognitive performance but also improves mood parameters and perception of stress. A dose response was demonstrated for Zynamite^®^ S, with the 100 mg dose suitable for moderate stress and cognitive demands and the 150 mg dose offering greater benefits, potentially suitable for more stressful and cognitively demanding situations. Overall, the findings support the acute dual cognitive and emotional benefits of 100 mg and 150 mg of water-soluble extract of *Mangifera indica* (60% mangiferin), Zynamite^®^ S. While the present study provides compelling evidence for the acute dual cognitive and emotional benefits of 100 mg and 150 mg of Zynamite^®^ S, further research is essential to fully elucidate its potential and address certain limitations. Future investigations should aim to mitigate the impact of the high inter-individual variability observed, perhaps by studying more homogeneous populations or employing designs that better control for physiological and psychological factors. Exploring the effects beyond a single administration is crucial to determine whether the observed benefits persist or accumulate with chronic supplementation. Additionally, testing a broader dose range would be valuable to identify optimal dosing strategies for different levels of cognitive demand or stress.

## Figures and Tables

**Table 1:** Preintervention sociodemographic and clinical characteristics of the participants as a whole and by group.

		Group A	Group B	Group C	*p*-Value
Age (years)		20.23 ± 1.48	20.31 ± 1.82	20.25 ± 1.48	0.973
Sex	Male (%)	21 (17.60)	19 (16.00)	20 (16.80)	0.943
	Female (%)	19 (16.00)	20 (16.80)	20 (16.80)	
Day	Morning (%)	30 (25.20)	29 (24.40)	30 (25.20)	0.997
	Afternoon (%)	10 (8.40)	10 (8.40)	10 (8.40)	
Sport	Yes (%)	30 (25.20)	30 (25.20)	24 (20.20)	0.318
	No (%)	10 (8.40)	10 (8.40)	15 (12.60)	
Height (m)		1.76 ± 0.09	1.75 ± 0.07	1.73 ± 0.09	0.680
Weight (kg)		73.11 ± 13.22	66.63 ± 13.13	66.05 ± 13.08	0.320
BMI (kg·m^2^)		23.45 ± 2.83	22.43 ± 5.46	22.05 ± 3.87	0.306
Waist (cm)		83.20 ± 9.38	78.28 ± 11.68	76.78 ± 9.92	0.170
Hip (cm)		100.95 ± 7.53	100.69 ± 11.59	96.90 ± 8.89	0.106
Relation waist/hip (—)		0.72 ± 0.06	0.78 ± 0.06	0.79 ± 0.05	0.200

Quantitative variables are presented as mean and standard deviation. Qualitative variables are presented as frequency and percentage. BMI: Body Mass Index; Zyn: Zynamite^®^; —: dimensionless quantity.

## Data Availability

The data presented in this study are available upon request from the corresponding author. The data are not publicly available because, due to the sensitive nature of the questions asked in this study, participants were assured raw data would remain confidential and would not be shared.
